# Complement as a Biomarker for Systemic Lupus Erythematosus

**DOI:** 10.3390/biom13020367

**Published:** 2023-02-15

**Authors:** Masahiro Ayano, Takahiko Horiuchi

**Affiliations:** 1 Department of Medicine and Biosystemic Science, Graduate School of Medical Sciences, Kyushu University, 3-1-1 Maidashi, Higashi-ku, Fukuoka 812-8582, Japan; 2 Department of Cancer Stem Cell Research, Graduate School of Medical Sciences, Kyushu University, 3-1-1 Maidashi, Higashi-ku, Fukuoka 812-8582, Japan; 3 Department of Internal Medicine, Kyushu University Beppu Hospital, 4546 Tsurumibaru, Tsurumi, Beppu 874-0838, Japan

**Keywords:** systemic lupus erythematosus, lupus nephritis, complement, split product, cell-bound complement activation product, serological activity

## Abstract

Systemic lupus erythematosus (SLE) is a disease of immune complex deposition; therefore, complement plays a vital role in the pathogenesis of SLE. In general, complement levels in blood and complement deposition in histological tests are used for the management of SLE. Thus, the evaluation of complement status can be useful in the diagnosis of SLE, assessment of disease activity, and prediction of treatment response and prognosis. In addition, novel complement biomarkers, such as split products and cell-bound complement activation products, are considered to be more sensitive than traditional complement markers, such as serum C3 and C4 levels and total complement activity (CH50), which become more widely used. In this review, we report the complement testing in the management of SLE over the last decade and summarize their utility.

## 1. Introduction

Systemic lupus erythematosus (SLE) is a typical systemic autoimmune disease characterized by the production of a variety of autoantibodies and the formation of immune complexes, with a chronic disease course and diverse organ involvement [1,2,3]. In 2014, besides the enhancement of SLE drugs, the treat-to-target concept was proposed for SLE [4], as previously proposed for rheumatoid arthritis. In addition, the basic framework of treatment strategies for SLE was established. In a treat-to-target strategy, treatment aiming at remission or where remission cannot be reached, the lowest possible disease activity must be conducted after periodically assessing overall SLE disease activity [4]. Furthermore, this strategy aims to ensure long-term survival, prevent organ damage, and optimize health-related quality of life by controlling disease activity and minimizing comorbidities and drug toxicity [4]. Therefore, these treatment strategies require strict management based on disease activity monitoring. Biomarkers can be used in this management, but they have not yet been established because SLE is associated with various organs with diverse immunological abnormalities [5,6,7].

SLE is a disease of immune complex deposition; thus, complements play a vital role in the pathogenesis of SLE [8,9,10]. In SLE, complements have a biphasic nature, which is known as the “lupus paradox [11].” That is, complement activation via immune complexes deposited in tissues causes tissue damage, whereas congenital deficiencies of the early components of complement activation pathways, such as C1q and C4, which are involved in the processing of apoptotic cells, frequently lead to the development of SLE [12]. In SLE, complement tests are usually abnormal, indicating complement-mediated pathologies. In general, complement levels in blood and complement deposition in histological tests are used for the management of SLE. Moreover, complement biomarkers can be useful in various settings, including the diagnosis of SLE, assessment of disease activity, and prediction of treatment response and prognosis.

This review outlines the reports of complement testing in the management of SLE over the last decade and summarizes its advantages. First, we discuss the advantages of serum C3 and C4 levels and total complement activity (CH50), the traditional complement markers, frequently used in routine clinical practice in various settings. Consequently, we also discuss novel biomarkers that are currently under research but are still expected to be applied and enhanced.

## 2. Overview on Complement

**Figure 1:**
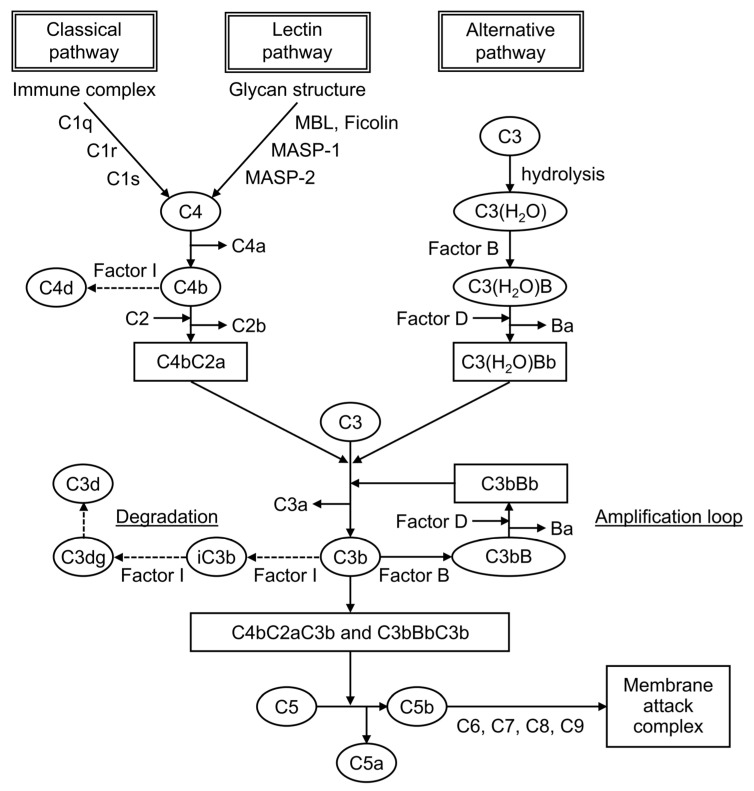
The complement cascade.

Complement is formed from more than 30 components found in blood and cell membranes, including plasma proteins, receptors on cell membranes, and regulatory factors. As shown in Figure 1, complement activation occurs through a series of chain reactions, which is known as a cascade. C3 activation is the most critical event. The complement response can be divided into two pathways: the “complement activation pathway” until C3 is degraded and the “late complement pathway” until the subsequent formation of the membrane attack complex. The complement activation pathway is divided into three: the classical pathway, the lectin pathway, and the alternative pathway. After C3 activation, the cascade to its deposition on a target as C3b and the formation of membrane attack complexes below C5 are common.

The factors that trigger the complement activation pathway are distinct, and they mediate different complement molecules. In the classical pathway, when an antibody binds to an antigen to form an immune complex, C1q binds to this immune complex and immediately activates C1r, which activates C1s. Then, this activated C1s activates C4 and then C2, leading to form C4bC2a, which is a C3-converting enzyme that activates C3. When C3 is activated, it is cleaved into fragments C3a and C3b, and C3b associates with C4bC2a to form C4bC2aC3b, a C5-converting enzyme that activates C5. It mediates the cleavage of the α-chain of C3b, leading to the formation of iC3b, followed by its further degradation into C3dg and C3d. In the lectin pathway, when mannose-binding lectin (MBL) and ficolin recognize and bind to the characteristic glycan structure of the pathogen cell wall, MBL-associated serine protease, a serine protease that is bound to MBL, is activated. Then, it cleaves C4 and C2 to form the C3-converting enzyme, which is similar to the classical pathway. In the alternative pathway, strictly speaking, no recognition molecule is found. C3(H_2_O), a hydrolyzed C3, is present in the blood, and it is activated when it binds to polysaccharides and other substances on the surface of microorganisms, binding an activated factor B and forming the C3-converting enzyme under the action of factor D, a serine protease.

Three pathways are involved in SLE, although the classical pathway is particularly important. Complement is not only a useful biomarker, as described below, but is also considered an important target for therapeutic drugs [13,14]. Currently, no approved complement-targeted therapies are available for SLE, but clinical trials are ongoing. Drugs in phase 2 trials for lupus nephritis include ravulizumab, an anti-C5 monoclonal antibody; iptacopan, a complement factor B inhibitor; vemircopan, a complement factor D inhibitor; narsoplimab, an anti-MBL-associated serine protease 2 monoclonal antibody; and pegcetacoplan, a PEGylated C3 inhibitor. In addition, KP104, a bifunctional biologic designed to simultaneously block both the alternative pathway (factor H) and the late complement pathway (C5), is in a phase 2 trial for thrombotic microangiopathies secondary to SLE.

## 3. Complement Tests

In routine clinical practice, serum C3 and C4 levels and CH50 are usually measured in blood samples, and complement deposition is identified by fluorescent antibody assay of tissues.

C3 and C4 are proteins of the complement cascade. In general, C3 and C4 are measured in serum, but samples such as urine, pleural fluid, and spinal fluid can also be used. In SLE, hypocomplementemia, decreased levels either of C3 and C4 or both occurs as an immunological abnormality (Table 1). C3 is included in the common cascade after the convergence of the three pathways. By contrast, C4 is included in the classical and lectin pathways. Therefore, low levels of both proteins indicate the activation of these two pathways, whereas low C3 and normal C4 indicate the activation of the alternative pathway. 

Plasma C4 levels are strongly influenced by and correlated with C4 gene copy numbers [15,16,17,18]. Each copy of the C4 gene encodes for one of two isotypes, acidic C4A or basic C4B. Congenital deficiency or a low copy number of the C4A gene has been associated with the development of SLE [19]. By contrast, a low C4B gene copy number did not show this association, and a medium to high C4B copy number was associated with thrombosis and hypertension in patients with pediatric SLE [15]. The profiles of C4 and C3 protein levels are different in each patient with SLE depending on the C4 gene copy number; therefore, there is a group of patients with low C4 levels due to low C4 gene copy number and without reflecting disease activity [16]. If only C4 shows persistently low levels, then the possibility of a low copy number of the C4 genes should be considered in the evaluation [16,17,18].

CH50 measures the hemolytic activity of serum samples against sensitized sheep erythrocytes. It is indexed by its ability to form membrane attack complexes via the classical pathway, and it reflects immune complex formation. It helps in complement-screening tests because it simultaneously measures all the complement activities from C1 to C9. In active SLE, CH50 levels are low because of complement consumption caused by the increased activation of the classical pathway (Table 1). If they are extremely low, then the possibility of a congenital defect is considered. In the case of low CH50 levels with normal C3 and C4 levels, it is important to exclude artificial in vitro complement activation (cold activation) after the blood sample is taken.

Complements are also used in the immunological testing of tissues. In SLE, reactions to C1q, C3, and C4 are observed in the renal glomeruli and at the dermo–epidermal border of the skin by fluorescent antibody assay. It is useful in evaluating affected organs and in the differentiation of other diseases.

Other novel biomarkers used include C1q, which is essential for the activation of the classical pathway; split product, which is a degraded product of complement; and cell-bound complement activation product (CB-CAP), a cell surface binding of C4d.

## 4. Traditional Complement Markers in SLE Diagnosis

### 4.1. SLE Diagnosis

The diagnosis of SLE has no gold standard; thus, it is based on classification criteria that reflect the variety of immunological abnormalities and diverse organ involvement characteristics of SLE. Although the SLE classification criteria have been revised in recent years, the currently used 2019 European League Against Rheumatism (EULAR) and American College of Rheumatology (ACR) classification criteria for SLE emphasizes immunological abnormalities and nephritis [20]. Consequently, complement, which was not included in the 1997 ACR classification criteria, was added to the list of immune domains in the current classification criteria, along with SLE-specific and antiphospholipid antibodies [20,21,22]. Hypocomplementemia is defined as a decrease in C3, C4, or CH50 based on the 2012 Systemic Lupus International Collaborating Clinics classification criteria, or low C3 and/or C4 based on the 2019 EULAR/ACR classification criteria [20,22]. A decrease in both C3 and C4, which suggests the involvement of the classical pathway, was considered as a more important finding, which was given a higher weight. Regarding the validation studies of the 2019 EULAR/ACR classification criteria, the frequency of hypocomplementemia (low C3 or C4) at diagnosis of SLE is 50%–89%, which is not commonly observed in all cases [23,24,25].

Among nephritis, class III or IV findings on renal biopsy are strongly suggestive of SLE, and in the new classification criteria, such findings can be classified as SLE along with immunological abnormalities [20]. Complement such as C1q, C3, and C4 deposition by immunofluorescence is not included in the definition of the pathologic classification of lupus nephritis [26]. It largely plays an ancillary role, but it is an important reference finding in differentiating lupus nephritis from other diseases [26,27]. SLE is characterized by C1q deposition because of the activation of the classical pathway and it often presents a “full-house pattern” with multiple deposits of immunoglobulins and complements. In addition to IgA nephropathy characterized by IgA deposition and anti-neutrophil cytoplasmic antibody-associated nephritis without immunoglobulin or complement deposition, C3 nephropathy with positive fluorescent findings of C3 deposition and negative or weak C1q, C4, and immunoglobulin deposition has recently been proposed [28]. Complement deposition in the fluorescent antibody assay is also useful in differentiating these renal diseases [29].

### 4.2. Diagnosis and Prediction of Organ Involvement

After the diagnosis of SLE, proper evaluation of the affected organs is important to provide optimal treatment. SLE presents with a variety of organ involvement, and it may be classified into several subgroups based on the organs affected and immunological abnormalities. Studies investigating the relationship between complements and organ involvement have shown the association of hypocomplementemia with nephritis, hematologic disorders such as autoimmune hemolytic anemia and thrombocytopenia, skin rash, and arthritis [30,31,32]. In a study of patients with SLE comparing low C4 levels alone with low levels of both C3 and C4, the frequency of serositis and hematologic disorders was lower, and the severity of renal and hematologic disorders was milder in the cases with low C4 levels alone [33].

The measurement of complements is also useful in appropriately ruling out other diseases to confirm whether organ involvement is caused by SLE. In a report in which renal biopsies were performed on 48 patients with SLE without abnormal urinalysis and renal impairment, 36 patients had lupus nephritis (class I or II, 72%; class III or IV, 17%; and type V, 11%), and hypocomplementemia (low titers of CH50 and C3) and a high titer of anti-Sm antibodies were identified as predictive factors for silent lupus nephritis. Complements measured in samples other than serum are used to differentiate other diseases [34]. Elevated levels of C3 in cerebrospinal fluid are useful in diagnosing neuropsychiatric SLE [35] and decreased levels of C3 and C4 in pleural fluid are useful in discriminating lupus pleuritis from pleural effusion of other etiologies [36].

## 5. Traditional Complement Markers in the Assessment of SLE Activity

In a treat-to-target strategy, SLE treatment is aimed at remission or, at least, low disease activity after regular evaluation of the overall disease activity [4]. Therefore, disease activity assessment indicators and treatment goals must be established.

In general, the assessment of disease activity in SLE is based on a combination of the type and severity of organ involvement and immunological abnormalities. Complements, along with anti-dsDNA antibody titers, are used in routine clinical practice as a biomarker for immunological abnormalities in SLE, and hypocomplementemia reflects the activity of SLE. The systemic lupus erythematosus disease activity index (SLEDAI), the validated activity index, frequently used for assessing global SLE activity, includes the presence of low complements, that is the decrease in CH50, C3, or C4 in qualitative assessment [37]. On the contrary, the British Isles Lupus Assessment Group index, which evaluates disease activity by each organ-based system, does not include immunological abnormalities [38]. In addition, the newly proposed SLE disease activity score includes swollen joint counts, proteinuria, leucocyte counts, and platelet counts as values in the score, but it does not include quantitative serological immune markers such as serum complement levels or anti-dsDNA antibody titers [39].

A composite definition is also used for low disease activity and remission, which are treatment targets of SLE. Although no criteria have been established, the lupus low disease activity state (LLDAS) is used as a general low disease activity criterion, and the definitions of remission in SLE (DORIS) is used as a remission criterion [40,41]. These composite indices combine the disease activity measured using the SLEDAI, the exclusion of any current disease activity not included in the SLEDAI, physician global assessment, and medication use, including glucocorticoid dosage. The definition of LLDAS allows for a SLEDAI-2K score of 4 or less. LLDAS may also be met, although hypocomplementemia and elevated anti-dsDNA antibodies remain unchanged. On the contrary, DORIS initially proposed complete remission, which requires no serological activities [42]. However, in the 2021 definition, remission will be evaluated by clinical SLEDAI-2K, which excludes immunological abnormalities from SLEDAI-2K, and serological activity is no longer an issue in the definition of remission [41]. Thus, whether treatment goals should include the normalization of immunological abnormalities remains debatable. Cases in which hypocomplementemia and elevated anti-dsDNA antibodies persist after achieving treatment goals such as low disease activity and remission are often observed. These cases of clinical remission with persistent immunological abnormalities have been recognized as serologically active clinically quiescent (SACQ) SLE [38]. The clinical significance of the SACQ status remains unclear, but a treat-to-target strategy and treatment recommendations do not recommend that the treatment in clinically asymptomatic patients is escalated primarily on the basis of stable or persistent serological activity [4,43,44].

Levels of complements must be evaluated regularly in routine clinical practice, along with symptoms, physical examination, and blood laboratory tests. Complement levels are not completely linked to SLE activity, as normal complement levels in the active phase and persistent hypocomplementemia in remission are often observed. SLE evaluation is based on global assessment; thus, levels of complements should not be overestimated but should be used as a guide for decision-making.

## 6. Traditional Complement Markers in Predicting SLE Treatment Response

In recent years, biologic drugs such as belimumab, an anti-B-cell activating factor (BAFF) monoclonal antibody, and anifrolumab, an anti-interferon-α receptor monoclonal antibody, have become available for SLE. Compared with glucocorticoids and conventional immunosuppressive drugs, which act on a relatively broad range of immune cells, biologic drugs have a clear therapeutic target, and the mechanism of action of the drug may predict treatment response. Belimumab is known to be more effective at high BAFF levels and anifrolumab at high interferon signatures [45,46]. However, these markers cannot be measured in routine clinical practice. Thus, markers predicting response to biologic drugs must be established within routine clinical practice. In this regard, low C3 levels and high anti-dsDNA antibody titers have been reported to be associated with high BAFF levels and interferon signatures [47,48]. In addition, belimumab and anifrolumab are more effective in patients with immunological abnormalities and are more likely to be effective in patients with high anti-dsDNA antibody titers, low C3 levels, and low C4 levels [46,49].

## 7. Traditional Complement Markers in Predicting SLE Prognosis

The prevention of flares is an important goal in the management of SLE. Early detection of signs of flares through careful monitoring of the global and organ-specific disease activity is essential, but understanding the risk factors that predict flares is also important. The 2019 update of the EULAR recommendations for the management of SLE identifies persistent serological activity (low complement and/or high anti-dsDNA antibodies) as risk factors, along with younger age at disease onset, no use of antimalarials, and persistent generalized disease activity [43].

In routine clinical practice, the assessment of the disease activity often considers the fluctuation in complement levels and anti-dsDNA antibody titers, but many clinical studies have reported the presence of hypocomplementemia at a single point in time, such as at the achievement of remission or at the beginning of observation. Gensous et al. and Kostopoulou et al. conducted a systematic literature review on whether complement predicts SLE flares and reported that many studies have shown that hypocomplementemia (low serum C3 and C4 levels) is a predictor of flares [50,51]. However, given the effects of differences in study methods, patient backgrounds, organ systems involved, and treatment, no firm opinion has been achieved. Regarding the persistent immunological abnormalities, patients with SACQ-SLE have a higher risk of flares than patients without immunological abnormalities [52,53,54], although few reports have focused on persistent hypocomplementemia. In addition, analyses focusing on fluctuations in complement levels have reported that progressive reduction in complement levels may indicate future flares in patients with SACQ-SLE [55].

Some reports have examined factors that predict the outcome for each organ involved rather than for SLE as a whole. In lupus nephritis, low serum C3 levels at diagnosis or at the time of remission may predict renal flares [56,57]. Patients with lupus nephritis and persistent isolated C3 hypocomplementemia were more likely to progress to end-stage kidney disease [58]. In patients with end-stage kidney disease, low serum C4 levels at the time of induction of renal replacement therapy were associated with SLE flares [59]. Severe neuropsychiatric manifestations and low serum C4 levels at the time of SLE diagnosis predict the development of severe neuropsychiatric flares [60]. A low serum C3 level could indicate complications of lupus enteritis [61,62], and a lower CH50 level at the time of initial treatment could predict an inadequate treatment response in patients with lupus enteritis [63]. Low serum C3 levels and high SLEDAI-2K scores were reported as independent risk factors for the development of lupus myocarditis in patients with SLE [64].

## 8. Novel Complement Biomarkers in SLE Management

### 8.1. C1q

C1q is essential for activating the classical pathway, and a decrease in C1q levels, as with C3 and C4 levels, indicates high disease activity in SLE (Table 1). Although the utility of C1q deposition in renal tissue and anti-C1q antibodies in the management of SLE is well established [65], serum C1q can also be a useful biomarker (Table 2). Serum C1q levels were decreased in patients with SLE compared with healthy controls, and they were associated with disease activity as measured by SLEDAI [66]. Serum C1q levels were also decreased in patients with lupus nephritis compared with healthy controls and patients with SLE uncomplicated by nephritis, indicating the activity of nephritis and the histological activity score of renal tissue [66,67,68].

### 8.2. Split Products

Split products are the cleavage fragments of the complement components. Known cleavage fragments of C3 include iC3b, C3dg, and C3d, and C4d is a cleavage fragment of C4 (Figure 1). The split products are only produced by complement activation, unaffected by increased production by the acute-phase response. Therefore, the assay is characterized by its ability to reflect complement activation in vivo more sensitively than the currently used C3 and C4.

iC3b is the breakdown product of C3b, a cleavage fragment of C3. Serum iC3b levels were elevated in patients with SLE compared with healthy controls, and the serum iC3b/C3 ratio was more sensitive to changes in disease activity than serum C3 or C4 levels, making it more useful for detecting active SLE and predicting flares [69]. Furthermore, iC3b is degraded to C3dg and C3d. Plasma C3dg levels were higher in patients with SLE than in healthy controls, and its discriminative power at the time of SLE diagnosis was superior to that of serum C3 [70]. Plasma and urine C3d levels were elevated in patients with active lupus nephritis and correlated with disease activity, and a decrease in values after treatment predicted subsequent treatment response [71].

C4d is a cleavage product of C4b produced from the degradation of C4. Plasma C4d levels were elevated in patients with SLE compared with healthy controls, and they were particularly high in patients with nephritis [72,73]. Plasma C4d levels correlated with C4d deposition in renal tissue, indicating nephritis activity, and they could be used in predicting flares and treatment response [73]. C5a is a cleavage product of C5. Recently, avacopan, a C5a antagonist, has proven to be effective in anti-neutrophil cytoplasmic antibody-associated vasculitis [74]. Plasma and urine C5a levels were elevated in patients with active lupus nephritis, and plasma C5a levels correlated with disease activity in lupus nephritis and SLE [75]. Bb is a cleavage product of factor B associated with the alternative pathway. Plasma Bb levels were elevated in patients with active lupus nephritis, indicating the activity score of the renal tissue and predicting poor renal prognosis [76].

Split products are considered superior biomarkers compared with serum C3 and C4. However, the stable measurement of such products is difficult because of their short half-life, and a simple and reliable measurement assay has not been established [8], although a novel, stable and simple assay using the iC3b/C3dg-binding site of human complement receptor 2 was reported [77].

**Table 2:** Summary of reports on novel complement biomarkers.

Reference	Author, Year	Sample	Number of Subjects	Key Findings
[66]	Sandholm, 2019	Plasma C1q	69 LN 310 SLE without LN 322 HC	SLE patients had lower levels of C1q than matched HCs (median, 225 vs. 266 mg/L, *p* < 0.001). An association was found between the levels of C1q and the SLEDAI. Patients with nephritis had lower levels of C1q than those without nephritis (median, 194 vs. 228 mg/L, *p* < 0.01).
[67]	Tan, 2013	Serum C1q	218 LN HC	The serum C1q levels were significantly lower in LN than those in HCs (33.8 ± 20.4 vs. 62.0 ± 10.5 μg/mL, *p* < 0.001). Patients with lower serum C1q levels (<40.97 μg/mL) showed significantly higher levels of SLEDAI (*p* < 0.001). The serum C1q levels were associated with renal total activity indices scores (rs = −0.327, *p* < 0.001).
[68]	Xu, 2019	Serum C1q	905 SLE without LN 334 active LN 255 inactive LN 497 HC	Significantly decreased C1q levels were observed in the active LN and inactive LN groups, which was in contrast to their levels in the SLE and HC groups (153.2 ± 40.0 vs. 170.8 ± 36.2 vs. 170.6 ± 35.5 vs. 182.3 ± 29.0 mg/L, *p* < 0.05).
[69]	Kim, 2019	Blood iC3b	159 SLE 48 HC	Patients with SLE had elevated iC3b levels as compared to HCs (4.5 ± 2.8 vs. 2.1 ± 0.9 μg/mL, *p* < 0.001). Patients with active SLE had elevated iC3b:C3 ratio as compared to patients with inactive SLE and HCs (7.0 ± 7.8 vs. 4.3 ± 2.0 vs. 1.7 ± 0.6 μg/mg, *p* < 0.001). The iC3b:C3 ratio correlated with the extent of SLE disease activity and with clinically meaningful changes in disease activity in patients with SLE. The iC3b:C3 ratio outperformed C3 and C4 levels with regard to discriminating active SLE from inactive SLE, and major flares from no disease activity.
[70]	Troldborg, 2018	Plasma C3dg	169 SLE 170 HC	SLE patients had higher concentrations in plasma C3dg than HCs (Data were presented only in figures, *p* < 0.001). ROC analysis showed that C3dg (AUC 0.96) was superior to C3 (AUC 0.52) in differentiating between patients and HCs.
[71]	Ganguly, 2020	Plasma C3d Urinary C3d	28 active LN 4 inactive LN 10 HC	Urine and plasma levels of C3d for active LN were significantly different from inactive LN (urine, median, 388 vs. 9.9 ng/mg Cr, *p* < 0.001; plasma, median, 791 vs. 212 μg/mL, *p* < 0.001). There was a significant correlation of plasma C3d with SLEDAI (rs = 0.67, *p* < 0.001) and renal SLEDAI (rs = 0.44, *p* = 0.03). There was a significant correlation of urine C3d with SLEDAI (rs = 0.433, *p* < 0.001) and renal SLEDAI (rs = 0.35, *p* < 0.001). Treatment responders at 6 months showed a significant fall in urine C3d at 3 months.
[72]	Martin, 2017	Plasma C4d	69 SLE 97 HC	C4d levels were negligible in HC subjects and significantly increased in patients with SLE (*p* < 0.001). C4d levels discriminated between higher and lower disease activity according to ROC curve analysis (AUC 0.64, *p* < 0.001). At higher disease activity, C4d levels correlated with the modified SLEDAI (rs = 0.26, *p* = 0.011) and predominantly with LN (*p* = 0.003).
[73]	Martin, 2020	Plasma C4d	22 SLE without LN 71 LN 145 HC	In comparison to HCs, plasma C4d levels were significantly increased in SLE patients (0.33 mg/L vs. 0.94 mg/mL, *p* < 0.001) with significantly higher levels in LN patients (1.02 mg/L) than in non-renal SLE patients (0.57 mg/L, *p* = 0.004). C4d levels correlated significantly with urine-albumin to creatinine ratio (rs = 0.43, *p* = 0.011), renal activity index (rs = 0.37, *p* = 0.002) and glomerular deposits of C4d in kidney biopsies (rs = 0.7, *p* = 0.0002). Plasma C4d declined significantly after treatment in patients that experienced favourable clinical and histopathological response (*p* < 0.0001), while levels remained mainly unchanged in non-responders.
[75]	Ma, 2018	Plasma C5a Urinary C5a	66 SLE 40 HC	Plasma C5a levels were dramatically elevated in patients with active LN compared to those in remission and HCs. Urinary C5a were significantly elevated in LN patients compared to HCs (*p* < 0.001). Correlation analysis showed that the plasma C5a levels were positively correlated with 24h proteinuria in LN patients (rs = 0.47, *p* = 0.002) and SLEDAI scores in SLE patients (rs = 0.28, *p* = 0.02).
[76]	Song, 2017	Plasma Bb, C1q, C4d, and C5a	82 SLE without LN 222 active LN 34 LN in remission 39 HC	Plasma Bb levels were significantly higher in patients with active LN compared with patients in remission, active SLE without LN, and HCs (1.24 ± 0.75 vs. 0.78 ± 0.45 vs. 0.90 ± 0.49 vs. 0.69 ± 0.45 μg/mL, *p* < 0.001). Plasma Bb level was significantly correlated with some renal disease activity indices and was a risk factor for renal outcomes (Hazard ratio = 1.75; 95% confidence interval = 1.106−2.754; *p* = 0.017) in the LN group.

LN, lupus nephritis; SLE, systemic lupus erythematosus; HC, healty control; SLEDAI, SLE disease activity index; ROC, receiver operating characteristic; AUC, area under the curve.

### 8.3. Cell-Bound Complement Activation Product (CB-CAP)

C4d is not only present in plasma, but it is also bound to the surface of blood cells. It is known as CB-CAP, which binds to erythrocytes, platelets, and B cells. C4d on each cell surface is measured using a flow cytometer. In 2004, erythrocyte C4d was measured and reported to be a biomarker with better sensitivity and specificity than traditional complement markers for the diagnosis of SLE [78]. It was later shown to be measurable on platelets, T cells, and B cells [79,80]. In the diagnosis of SLE, erythrocyte C4d and B-lymphocyte C4d could be used to observe elevated C4d on the surface of each cell, even in cases negative for anti-dsDNA antibodies [81]. Thus, a multianalyte assay panel, commercially known as the AVISE Lupus test (Exagen Inc., Vista, CA, USA), which combines erythrocyte C4d and B-lymphocyte C4d with eight lupus and non-lupus autoantibodies, has recently been used in distinguishing SLE from a variety of other rheumatic diseases [82,83]. In monitoring SLE activity, C4d on each cell surface was more sensitive and specific than serum C3 and C4, which acutely reflected activity even in cases with normal or no fluctuation in serum C3 and C4 levels [84,85]. In addition, platelet C4d was a useful marker for predicting thrombosis and vascular events [86,87,88].

C4d also deposits in renal tissue. Strong C4d deposition was consistently present in immune-complex glomerulonephritis, including lupus nephritis [89], and C4d deposition in renal peritubular capillaries is an important finding in the diagnosis of antibody-mediated rejection in kidney transplantation [90]. C4d deposition was common in the renal tissues of patients with lupus nephritis [91,92]. These deposits were localized in the glomeruli in almost all cases, followed by tubular basement membrane, arterioles, and peritubular capillaries [92]. Tubular basement membrane C4d deposition was related to the disease activity, and arteriolar C4d deposition was associated with worse renal outcomes [92].

## 9. Conclusions

This study summarized the usefulness of complement as a biomarker in the SLE clinical practice. Serum C3 and C4 levels, which are frequently used in routine clinical practice, are included in the classification criteria for SLE and disease activity indices. They are also considered useful SLE biomarkers. However, they are not absolute indicators that can be determined by complement alone. Therefore, the characteristics and limitations of the complement test must be fully understood and comprehensively evaluated in conjunction with clinical signs and anti-dsDNA antibody titers. The complement split products and CB-CAP might reflect SLE activity more sensitively than traditional complement markers and are expected to become more widely used.

## Figures and Tables

**Table 1:** Complement markers.

Complement Markers	Abnormalities Observed in Patients with SLE	Complement Pathway Involved		
		Classical	Lectin	Alternative
Functional assay				
CH50	low	+	–	–
Complement components				
C3	low	+	+	+
C4	low	+	+	–
C1q	low	+	–	–
Complement split products				
iC3b	high	+	+	+
C3dg	high	+	+	+
C3d	high	+	+	+
C4d	high	+	+	–
C4a	high	+	+	–
C5a	high	+	+	+
Bb	high	–	–	+
